# Epidemiology of Coccidioidomycosis in the Veterans Health Administration, 2013–2022

**DOI:** 10.3390/jof9070731

**Published:** 2023-07-06

**Authors:** Cynthia Lucero-Obusan, Rishi Deka, Patricia Schirmer, Gina Oda, Mark Holodniy

**Affiliations:** 1 Public Health National Program Office, Department of Veterans Affairs, Washington, DC 20420, USA; 2 Division of Infectious Diseases and Geographic Medicine, Stanford University, Stanford, CA 94305, USA

**Keywords:** coccidioidomycosis, Valley Fever, *Coccidioides*, epidemiology, mycotic diseases, fungal diseases, endemic mycoses, respiratory infections, Veterans

## Abstract

We describe the epidemiology of coccidioidomycosis among a national cohort of Veterans. Using electronic health record data from adults tested for coccidioidomycosis between 1 January 2013 and 31 December 2022, we analyzed differences in baseline demographics (age, sex, race/ethnicity, birth country, comorbidities, residence, and Charlson Comorbidity Index score) between 4204 coccidioidomycosis-test-positive and 63,322 test-negative Veterans. Log-binomial regression models with adjusted risk ratios (aRRs) were used to evaluate risk factors associated with coccidioidomycosis including dissemination, hospitalization, and mortality. Case counts and incidence rates were highest in select counties in Arizona and California where *Coccidioides* is endemic. Coccidioidomycosis-positive Veterans were younger, more likely to be male, and Philippine-born. The risk factors most highly associated with being coccidioidomycosis-positive included Native Hawaiian/Pacific Islander (aRR 1.068 [95%CI: 1.039–1.098]), Asian (aRR 1.060 [95%CI: 1.037–1.083]), Black (aRR 1.029 [95%CI: 1.022–1.036]), American Indian/Alaska Native (aRR 1.026 [95%CI: 1.004–1.048]) race, and Hispanic/Latino ethnicity (aRR 1.021 [95%CI: 1.013–1.028]). Black race (aRR: 1.058 [95%CI: 1.037–1.081]) and Hispanic/Latino ethnicity (aRR 1.018 [95%CI: 1.0003–1.036]) were also associated with disseminated coccidioidomycosis, strengthening the evidence for the association of coccidioidomycosis, including severe infections, with specific racial and ethnic groups. There were no statistically significant differences in hospitalization within 45 days of testing or 30-day all-cause mortality. Improving our understanding of coccidioidomycosis risk factors is important for targeted prevention strategies and to reduce delays in diagnosis and ineffective treatment.

## 1. Introduction

Coccidioidomycosis (Valley Fever) is a fungal infection caused by the endemic, soil-dwelling fungus *Coccidioides* spp., being of particular importance in the Western and Southwestern United States (US), as well as parts of Mexico and Central and South America [1,2,3,4]. Coccidioidomycosis is most often characterized by a self-limited respiratory illness or pneumonia but may progress to more severe pulmonary disease or a disseminated infection. Patients often present 1–3 weeks following exposure to airborne arthroconidia with symptoms that may include fever, cough, shortness of breath, headache, chills, night sweats, weight loss, fatigue, and/or joint pain [3,4]. Infections often mimic other types of community-acquired pneumonia, leading to a lack of clinical suspicion for coccidioidomycosis and underutilization of provider-directed laboratory testing [3,4,5]. In some cases, this leads to significant delays in diagnosis and/or inappropriate treatment, including antibacterial agents [6,7]. The epidemiology of coccidioidomycosis in the US has changed due to expansion of the geographic range of the fungus, likely related to climate change and other environmental factors, resulting in outbreaks and increased incidence in recent years [2,3,4,8,9,10,11,12,13,14]. Coccidioidomycosis results in substantial morbidity and is a disease of increasing public health importance, with approximately 20,000 cases reported annually to the US Centers for Disease Control and Prevention (CDC) [15]. This is likely to be a significant underestimate, due to misdiagnosis, undertesting, and underreporting, such that true disease burden is estimated to be 6–14 times higher than reported cases, in the range of 100,000–350,000 new coccidioidomycosis infections in the US annually [1,2,5,7,16].

Most infections in the US are believed to be acquired in southern Arizona, central and other parts of California, Nevada, southern New Mexico, Utah, Washington, and west Texas [3,4,9,17]. The majority of coccidioidomycosis infections are mild/asymptomatic and self-limiting, resolving within a few weeks to months after symptom onset and often without requiring treatment with antifungal agents [3,4]. However, previous studies have suggested that the risk of complications and/or severe infection is higher among men, immunocompromised individuals, pregnant women, Black individuals, those with Filipino ancestry, and patients with diabetes [1,18,19]. Severe infections may include complicated pulmonary infections and disseminated disease involving the skin, bones, joints, and/or the central nervous system (such as meningitis) [3,4]. Severe and/or refractory infections may necessitate hospitalization of individuals with coccidioidomycosis, and in some instances may result in death. Each year, coccidioidomycosis is estimated to cause approximately 160–200 deaths and 2000–3000 hospitalizations in the US [1,20,21]. Large-scale, systematic, laboratory-confirmed evaluations assessing the effect of coccidioidomycosis leading to severe outcomes, such as hospitalization and mortality, are warranted to further characterize the burden of disease and inform prevention efforts.

The objective of this study was to characterize the epidemiology of laboratory-confirmed coccidioidomycosis infections, including risks for severe infection, hospitalization, and mortality, among a national cohort of US Veterans. US Veterans represent a potentially high-risk population for severe coccidioidomycosis infections given most enrollees in US Department of Veterans Affairs (VA) healthcare are males with higher rates of underlying comorbidities [22,23] and a greater potential for environmental dust exposure during military service and desert training exercises [24]. Prior studies among US Veterans during the 1950–1960s provide historical context for the natural history of coccidioidomycosis, including disseminated infections in the US Veteran patient population, but more recent patient data are lacking [25,26,27]. Strengthening the evidence base for the association of coccidioidomycosis infections with severe morbidity outcomes will help guide prevention, testing, treatment, and control efforts, including potential future vaccines for coccidioidomycosis.

## 2. Materials and Methods

### 2.1. Study Population, Data Sources, and Procedures

The Veterans Health Administration (VHA) is the largest integrated healthcare system in the US, providing care to approximately 6.75 million patients across 1321 care sites nationwide [28]. VHA inpatient and outpatient medical encounters are maintained in a nationwide database of electronic health records (EHRs), known as Veterans Information Systems and Technology Architecture (VistA). EHR data were obtained from the Corporate Data Warehouse (a repository and health data warehouse comprising VistA clinical data and other data systems) and VHA’s Public Health National Program Office’s Praedico™ Surveillance System (Bitscopic, Los Angeles, CA, USA), which compiles public health data across multiple EHR domains, including diagnostic testing. For our analysis, we included adults (age ≥ 18 years) who were tested for coccidioidomycosis during VHA care between 1 January 2013 and 31 December 2022. Individuals under age 18, non-Veterans (including those on active duty and dependents, non-Veteran employees, and others with non-Veteran eligibility status), those with scanned test result reports that could not be retrieved electronically, and those with indeterminate, equivocal, or anticomplementary results were excluded.

Designation as coccidioidomycosis-positive was based on the current Arizona and California laboratory criteria for a confirmed case, defined as a single positive coccidiomycosis result including *Coccidioides* spp. identified via fungal culture or histopathology; molecular assay (including PCR, DNA probe, or MALDI-TOF); antigen; enzyme immunoassay (EIA/ELISA); immunodiffusion; complement fixation (CF); latex agglutination; tube precipitin; or lateral flow assay [29,30]. Patients were considered coccidioidomycosis-positive based on the laboratory-only surveillance case definition if at least one of the above test results were positive, even if specimens were collected outside of Arizona and California. Patients were considered negative if all available coccidioidomycosis testing performed was negative. For patients with multiple coccidioidomycosis diagnostic tests during the review time frame, we used the earliest positive test result for cases and earliest negative test result for patients where all coccidioidomycosis testing was negative. Annual state and county rates for coccidioidomycosis positivity were calculated per 100,000 Veterans in care, based on the VHA Support Service Center Capital Assets (VSSC) Unique Patients data cube by the estimated date of onset, utilizing the earliest positive coccidioidomycosis test result.

Covariates of interest extracted from the EHRs included age, sex, race, ethnicity, country of birth, county of residence at time of coccidioidomycosis testing, and underlying comorbidities. Comorbidities were based on the *International Classification of Diseases, Clinical Modification, 9th and 10th Revision* (ICD-9 and ICD-10) codes pulled from 2010–2022 inpatient and outpatient medical records that occurred prior to the index coccidioidomycosis test collection date or from conditions listed on the electronic problem list at any time prior to the test collection date. ICD codes were then grouped into major categories based on the Charlson Comorbidity Index (CCI) classifications, and into other relevant conditions based on Healthcare Cost and Utilization Project (HCUP) clinical classification software (CCS) [31,32]. The CCI is a weighted summary score of the quantity and severity of comorbidities that has widely demonstrated associations with health outcomes both in the general population as well as the VHA patient population [33,34]. Because military training locations and deployment locations were not electronically available from VHA records, we performed chart reviews for all coccidioidomycosis individuals <25 years of age at the time of initial testing to determine if military training and deployment information is routinely recorded by providers and whether any Veterans in this youngest age cohort were first diagnosed during active-duty military service. For the purposes of this study, coccidioidomycosis-negative individuals with a diagnosis code or problem list entry for an invasive fungal infection [35] or a diagnosis code for coccidioidomycosis without a positive test result in the VHA during the time period analyzed were excluded, due to the potential for confounding in this analysis.

### 2.2. Statistical Analysis

The distributions of demographic characteristics (age, sex, race, ethnicity, birth country, and county of residence) as well as comorbidities at the time of coccidioidomycosis testing were compared between patients who tested positive for coccidioidomycosis and those who tested negative using Pearson’s chi-square test for categorical variables and the Mann–Whitney–Wilcoxon (MWW) test for continuous variables. County incidence rates for coccidioidomycosis were based on 2021 county annual incident rates for California [36] and Arizona [37] and 2011–2017 rates reported by CDC for the remainder of the US [9]. Using published rates from these sources, counties were grouped into categories of “high incidence rate” (≥100 per 10,000 population), “medium incidence rate” (6–99.9 per 100,000 population), and “low incidence rate” (<6 per 100,000 population) for the purposes of this analysis. Log-binomial regression models with adjusted risk ratios (aRRs) were used to evaluate the association of various risk factors with a positive binary coccidioidomycosis test or binary disseminated coccidioidomycosis (including meningitis). Regression models were adjusted for variables including age, sex, race, ethnicity, specific comorbidities, CCI score, and birth country. Individuals were considered to have disseminated coccidioidomycosis (including meningitis) if they had an inpatient or outpatient encounter between 1 January 2013 and 31 January 2023, or a problem list entry with an ICD-9/ICD-10 code of 114,2, 114,3, B38.4, or B38.7. Outcomes assessed in this analysis were all acute hospitalizations within 45 days before or after the date of specimen collection for earliest coccidioidomycosis diagnostic testing, all coccidioidomycosis-coded outpatient encounters, and hospitalizations for test-positive patients between 1 January 2013 and 31 January 2023, and all-cause mortality within 30 days following coccidioidomycosis diagnostic testing. We included all hospitalizations within 45 days of testing date due to multiple reports describing significant delays in coccidioidomycosis diagnosis, with 43–54% of patients having a diagnosis delay >1 month, and in other studies, the median time from symptom onset to diagnosis ranged from 38 to 55 days [38,39,40,41]. Log-binomial regression models with adjusted risk ratios were also used to evaluate the association of coccidioidomycosis positivity with acute care hospitalization and all-cause mortality. A *p*-value of 0.05 was set as the cutoff for statistical significance. Statistical analysis was performed using R version 4.2.2 (R Foundation for Statistical Computing, Vienna, Austria).

### 2.3. Ethics Statement

The data utilized in this study were obtained for the purpose of public health operations in the VHA. No additional analyses outside of public health operational activities were performed. Therefore, this study was deemed to meet the requirements of public health surveillance as defined in 45 CFR 46.102(I)(2). This project was approved by the Stanford University Institutional Review Board (Protocol ID 47191, “Public Health Surveillance in the Department of Veterans Affairs”) and written informed consent was waived.

## 3. Results

### 3.1. Study Population and Descriptive Characteristics

From 1 January 2013 to 31 December 2022, 78,818 adults (age range 18–105 years) had 251,577 records for coccidioidomycosis diagnostic testing within the VHA system. The number of tests performed increased over time during the study period, from 17,061 in 2013 to 36,445 in 2022. Testing was primarily ordered at VHA facilities in AZ (119,847; 47.6%), CA (71,439; 28.4%), TX (17,567; 7%), NV (8153; 3.2%), MI (2798; 1.1%), IN (2762; 1.1%), OH (2248; 0.9%), and NM (2215; 0.9%). Females accounted for 6756 (8.6%) of those tested. The median age at time of testing was 66 years (IQR: 56–73 years). Of the tested adults, 11,292 were excluded from further analysis due to a non-Veteran status, equivocal/indeterminate/anticomplementary coccidioidomycosis test results, the absence of electronic test results, the presence of a recorded diagnosis of another invasive fungal infection, or a diagnosis code for coccidioidomycosis without a positive test result in the VHA during 2013–2022. This left 63,322 coccidioidomycosis-test-negative patients and 4204 patients with one or more positive test for coccidioidomycosis, which were included in subsequent analysis.

**Figure 1:**
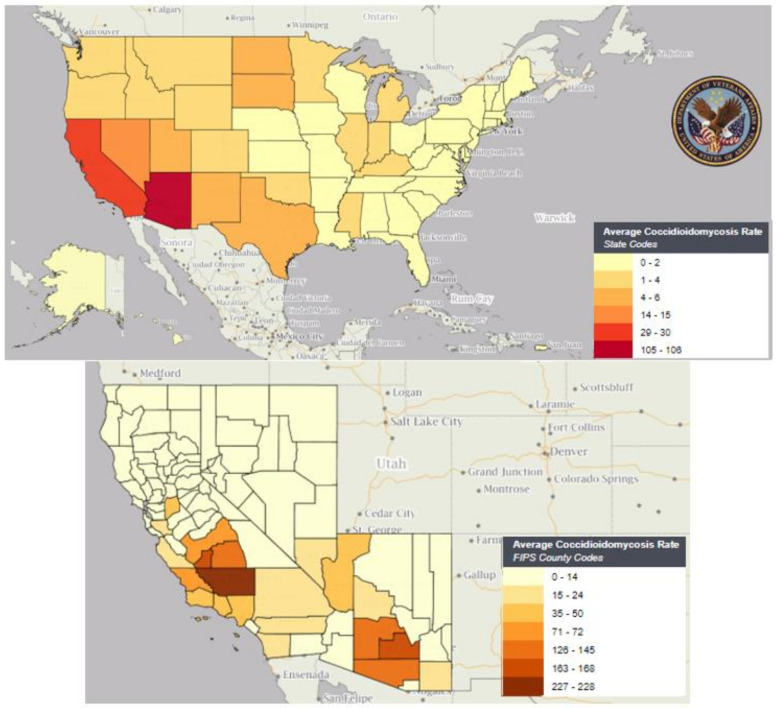
Average coccidioidomycosis-positive rate per 100,000 Veterans in care, by state (upper panel) and select counties (lower panel), Veterans Health Administration, 2014–2022. Crude rates were calculated per 100,000 Veterans in care by state and county (based on Core Facility Uniques in VSSC Unique Patients Data Cube, Core Facility Uniques) by estimated date of onset using the earliest positive coccidioidomycosis test date. AZ, CA, and NV counties with at least 1 VHA coccidioidomycosis-positive case per calendar year or 4 or more positive cases in a single calendar year are displayed. All other counties are shown in the lightest color as rates are potentially unreliable due to low case counts. Veterans in care are tabulated by VHA fiscal year (1 October to 30 September each year).

Patient states of residence at the time of earliest positive testing during 2013–2022 were AZ (1760; 41.9%), CA (1382; 32.9%), TX (259; 6.2%), NV (117; 2.8%), WA (42; 1%), and other states (686; 16.3%). The counties with the highest number of coccidioidomycosis-positive cases from 2013 to 2022 were Maricopa, AZ (915; 21.8%), Pima, AZ (524; 12.5%), Los Angeles, CA (294; 7.0%), Kern, CA (194; 4.6%), Pinal, AZ (180; 4.3%), San Diego, CA (166; 3.9%), Fresno, CA (113; 2.7%), Clark, NV (100; 2.4%), Tulare, CA (86; 2.0%), and Riverside, CA (77; 1.8%). Crude rates for coccidioidomycosis-positive per 100,000 Veterans in care for 2014–2022 based on estimated date of onset were compared to published rates for Arizona and California [36,37]. The highest rates (average rate greater than 100 per 100,000 Veterans in care) were observed in Kern County, CA (average: 227.7, range 33.5–372.0); Pinal County, AZ (average 168.0, range 116.6–280.5); Kings County, CA (average 163.1, range 74.5–268.3); Pima County, AZ (average 144.5, range 88.8–172.4); Tulare County, CA (average 144.4, range 70.8–280.2); and Maricopa County, AZ (average 126.2, range 89.6–186.9) (Table 1/Figure 1).

Basic demographic comparisons for coccidioidomycosis-positive and coccidioidomycosis-negative patients were calculated (Supplementary Figure S1). The median age (64 years, IQR: 54–72, range 20–100 years) for patients testing positive for coccidioidomycosis was younger than those who were negative. More than half (2106; 50.1%) were aged 65+ at the time of initial positive testing, with less than 1% being in the 18–24 years age group (23; 0.5%). Chart reviews for all 23 patients in the 18–24 years age group found that 19/23 (82.6%) had at least some documentation by VHA providers or DoD health records of their military training and/or deployment locations. Seven (30.4%) were first diagnosed with coccidioidomycosis or had documented presumed exposures during active duty, including six with meningitis or disseminated coccidioidomycosis. These exposures occurred at Fort Irwin National Training Center in San Bernardino County, CA (n = 2); Naval Air Station Lemoore in Fresno and Kings County, CA (n = 2); Marine Corps Base Camp Pendleton in San Diego County, CA (n = 1); Marine Corps Air Station in Yuma County, AZ (n = 1); and a family visit to Bakersfield, Kern County, CA, prior to deployment in Oslo, Norway (n = 1).

A higher percentage of individuals positive for coccidioidomycosis were male sex, Black, Asian, American Indian/Alaska Native race, and Hispanic/Latino ethnicity compared to those testing negative (Table 2). Being born in the Philippines or residing in a US county with a medium or high incidence of coccidioidomycosis was also significantly higher in the positive group. There was a high prevalence of underlying medical conditions, with more than one-quarter of patients in both groups having a CCI severity score of 5 or greater. Among all patients, however, the median CCI score was higher in the coccidioidomycosis-negative group. The occurrence of certain medical conditions, such as malignancy, renal disease, chronic obstructive pulmonary disease (COPD), hemiplegia/paraplegia, rheumatic disease, dementia, and certain cardiac and vascular diseases, was more common among those testing negative for coccidioidomycosis.

### 3.2. Risk Factors Associated with Coccidioidomycosis and Disseminated Coccidioidomycosis

Risk factors having the highest association with being coccidioidomycosis-positive compared to coccidioidomycosis-negative were age, Native Hawaiian/Pacific Islander, Asian, Black, or American Indian/Alaska Native race, unknown race, and Hispanic/Latino ethnicity (Table 3). None of the comorbidity groupings found to be associated with coccidioidomycosis in prior studies were significantly associated with coccidioidomycosis positivity in our cohort. Being of Black race (aRR: 1.058 [95% CI:1.037, 1.081]) or Hispanic/Latino ethnicity (aRR: 1.018 [95% CI: 1.0003–1.036]) was associated with a slight, but statistically significant increased risk of disseminated coccidioidomycosis compared to non-disseminated coccidioidomycosis (Table 4).

### 3.3. Hospitalizations, Outpatient and Emergency Department Encounters, and All-Cause Mortality

One thousand four hundred and ninety-nine (35.6%) patients who tested positive for coccidioidomycosis had any acute care hospitalization within 45 days before or after their initial positive coccidioidomycosis test compared to 24,272 (38.3%) patients who tested negative for coccidioidomycosis. Additionally, 109 (2.5%) patients who tested positive for coccidioidomycosis and 2428 (3.8%) who were coccidioidomycosis-negative died within 30 days of their earliest coccidioidomycosis test date. There were no statistically significant associations between those who were positive for coccidioidomycosis versus negative in terms of hospitalizations within 45 days of testing or 30-day all-cause mortality (Table 5). Among the patients who tested positive for coccidioidomycosis, 762 (18.1%) had a total of 1629 ICD 9/10-coded coccidioidomycosis hospitalizations between 1 January 2013 and 31 January 2023, with a median length of stay of 4 days (range 1–243 days), and 82/762 (10.8%) patients died during a coccidioidomycosis-coded hospitalization. Coccidioidomycosis-coded hospitalizations primarily occurred in VHA facilities in AZ (788; 48.4%), CA (518; 31.8%), TX (68; 4.2%), NV (42; 2.3%), and FL (21; 1.3%). A total of 422 (25.9%) of these 1629 hospitalizations included a diagnosis code of disseminated coccidioidomycosis (ICD 9/10: 114.3/B38.7) and/or coccidioidomycosis meningitis (ICD 9/10: 114.2/B38.4).

**Figure 2:**
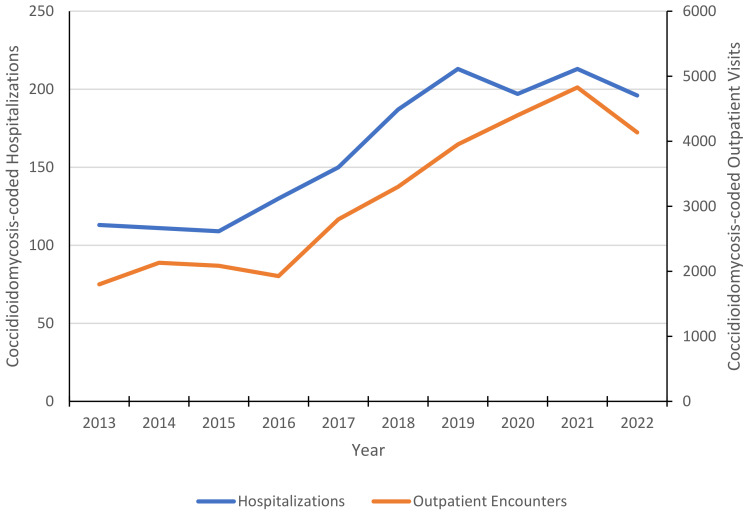
Annual number of ICD 9/10-coded coccidioidomycosis outpatient visits and hospitalizations among coccidioidomycosis-positive patients, Veterans Health Administration, 2013–2022. Includes coccidioidomycosis-coded encounters and hospitalizations for all test-positive patients by year, regardless of the date of diagnosis.

From 1 January 2013 to 31 January 2023, there were 31,642 coccidioidomycosis-coded outpatient encounters (including telehealth visits) among coccidioidomycosis-positive individuals, including 12,126 (38.3%) in infectious diseases, 7211 (22.8%) in primary care/medicine, 3797 (12%) in pulmonary, 803 (2.5%) in physical medicine and rehabilitation/physical therapy/occupational therapy/kinesiotherapy, 646 (2%) in rheumatology, and 312 (1%) were coccidioidomycosis-coded during emergency department/urgent care visits. Most coccidioidomycosis-coded outpatient visits occurred at VHA facilities in CA (12,890; 40.7%), AZ (10,621; 33.6%), TX (1535; 4.9%), NV (971; 3.1%), and NM (652; 2.1%). There was an average of 7.5 coccidioidomycosis-coded outpatient visits per coccidioidomycosis-positive patient. The annual number of coccidioidomycosis-coded hospitalizations and outpatient visits among test-positive individuals increased over the study period (Figure 2).

## 4. Discussion

Among a large national cohort of patients who tested for coccidioidomycosis within the VHA, we found that Veterans who were coccidioidomycosis-positive were more likely to be younger and male. Despite this, the median age for coccidioidomycosis positivity was 64 years. The predominance of cases in older adults and males in our patient population is consistent with prior reports in the general population [2,8,9]. Philippine-born, Hispanic ethnicity, and Black, Asian, Native Hawaiian/Pacific Islander, and American Indian/Alaskan Native race were also associated with coccidioidomycosis positivity in Veterans. Additionally, for disseminated coccidioidomycosis, Black race and Hispanic/Latino ethnicity were significant risk factors. These findings reinforce existing evidence that coccidioidomycosis, including severe infections, is associated with specific racial and ethnic groups [3,4,6,8]. Although the precise reasons behind racial differences in coccidioidomycosis acquisition and severity are unclear and likely multifactorial, factors including socioeconomic, social determinants of health, health disparities, access to healthcare, higher rates of underlying comorbidities, occupations, immunologic mechanisms, and genetic predisposition may all play a role [3,42]. When looking at specific comorbidities, diabetes, HIV/AIDS, immunity disorders, and mild and moderate/severe liver disease were more common among coccidioidomycosis-positive patients, but the differences did not reach statistical significance. An association of coccidioidomycosis with diabetes, HIV/AIDS, and immunity disorders has been documented previously [2,3,4,5,19]. Two recent case series have also described severe or disseminated coccidioidomycosis in the setting of cirrhosis and end-stage liver disease [43,44]. It is unclear why other comorbidities (including malignancy, renal disease, COPD, hemiplegia/paraplegia, rheumatic disease, dementia, and certain cardiac and vascular diseases) were more common in the coccidioidomycosis-negative group. The test-negative group was in general older (median age 66 years versus 64 in the test-positive group), which may explain why they had more comorbidities and a higher median CCI score.

Our review found that the increasing testing, outpatient visits, and hospitalizations for coccidioidomycosis in the past decade are likely reflective of an increased prevalence of coccidioidomycosis among VHA patients due to increased awareness among providers, accessibility of tests and testing modalities, and identification of coccidioidomycosis in Veteran patients. Encounter and hospitalization increases are also likely impacted by provider coding practices and the need of some patients with coccidioidomycosis for serial testing, long-term treatment, and/or follow-up over months to years. High crude incidence rates among VHA enrollees in select counties in Arizona and California aligned closely with those counties known to have a high incidence of coccidioidomycosis based on state surveillance data. Rates in the VHA may differ from published rates due to the lack of pediatric patients, for which documented rates of coccidioidomycosis are lower, and the older average age of those tested for coccidioidomycosis in the VHA, who may have fewer outdoor occupational exposures and are far removed in time from military training and deployments. Variability in rates by geographic region over time may be impacted by environmental factors (such as weather, precipitation, and drought); natural disasters (including wildfires, earthquakes, landslides, dust storms); population dynamics (resulting in residential and other construction); and other soil disturbances and dust-producing activities, as suggested in previous regional analyses [2,3,4,12,13,14].

As a large, national health system, we were able to identify cases across the US and US territories, including states and jurisdictions where coccidioidomycosis is not a reportable condition and surveillance data are limited or lacking [45]. Although testing in the VHA increased during the study period, VHA providers are likely still undertesting for coccidioidomycosis. A recent study in one VHA facility highlighted the prevalence of fungal pathogens, including coccidioidomycosis, in hospitalized Veterans with acute respiratory illnesses (ARIs). In this study, 13% of hospitalized Veterans in Houston (a region of Texas where coccidioidomycosis is not thought to be common) with ARIs and negative viral respiratory testing tested positive for coccidioidomycosis [46]. In the coming years, coccidioidomycosis is likely to further increase in incidence due to climate change and the expansion of the geographic range of the *Coccidioides* fungus [13,14]. Continued emphasis on targeted educational campaigns is needed to increase awareness and testing for coccidioidomycosis among higher risk groups, including within the VHA.

This study was subject to several limitations. Our data source does not contain laboratory results from non-VHA facilities or clinical records for patients who received care in non-VHA facilities unless these services were paid for by VHA. Therefore, coccidioidomycosis testing, outpatient ICD 9/10-coded visits, and some hospitalizations falling into this category were not included in our study. Additionally, we were unable to access EHR data from five VHA facilities after their migration from VistA to the Cerner EHR system. These facilities include Spokane, WA (as of 24 October 2020); Walla Walla, WA (as of 26 March 2022); Columbus, OH (as of 30 April 2022); Roseburg and White City, OR (as of 11 June 2022). Data prior to the migration were included (a total of six coccidioidomycosis-positive cases from Spokane and one each from Roseburg and White City). Given the small number of cases recorded by these facilities, we do not anticipate that having the post-migration data would change results significantly, although we expect these data will be available to the VHA Public Health National Program Office in the future. Males are overrepresented in the VHA patient population and our dataset, which may have impacted our power to analyze sex as a risk factor, including for disseminated coccidioidomycosis, as seen in prior studies [8,24,41,47]. Some patients with mild or asymptomatic coccidioidomycosis infections may never seek care or undergo testing and therefore would not be captured by our methods. We did not assess whether test-positive patients received antifungal treatment, nor the duration of treatment or potential effects of treatment on outcome. Disseminated coccidioidomycosis was based on assigned diagnosis codes and problem lists. We did not perform chart reviews to confirm the accuracy of coding for disseminated disease or review records with an assigned coccidioidomycosis diagnosis code where test results were not electronically available. We also suspect that miscoding and undercoding may be occurring due to the complexity in interpreting coccidioidomycosis test results. Additionally, some coccidioidomycosis test results were not available electronically, but were inserted in the medical record as a scanned image. For individuals where subsequent positive coccidioidomycosis testing was identified, we did perform chart reviews for the prior scanned laboratory reports to determine the earliest date on which they tested positive. However, we excluded individuals where there was no other positive coccidioidomycosis testing, since we were unable to perform chart reviews for all scanned laboratory reports. Due to these reasons, our data likely underestimate the true burden of coccidioidomycosis among VHA enrollees.

For this study, we utilized a streamlined laboratory case definition and reported coccidioidomycosis-positive cases rather than classifying individuals as confirmed, probable, or suspected cases as per the 2023 CSTE coccidioidomycosis case definition [48]. We did not collect evidence needed to establish clinical criteria or epidemiologic linkage for individuals in counties with an incidence of <10 cases per 100,000. Arizona and California currently use a laboratory-only case definition, which does not require clinical or epidemiologic linkage, and the majority of our coccidioidomycosis-positive cases came from these jurisdictions. For geographic classification, we used patients’ county and state of residence at the time of their first positive coccidioidomycosis test. It is expected that some cases are travel-related, and exposures occurred outside of the recorded residence. For example, slightly higher rates seen in states such as Minnesota and North and South Dakota likely represent a snowbird phenomenon for individuals who reside part of the year in a *Coccidioides*-endemic area but whose address of record and/or VHA facility where they receive care is outside an endemic area. We were not able to extract military training and deployment histories electronically from VHA medical records. However, by using chart reviews for 23 coccidioidomycosis-positive individuals who were under 25 years of age at the time of initial testing in the VHA and closest in time from their active-duty service, we found that more than 25% had documented or presumed exposures at military installations in coccidioidomycosis-endemic regions of California or Arizona, including six with disseminated coccidioidomycosis or meningitis. This supports previous studies that documented seroconversions and disseminated coccidioidomycosis among military service members with service time in these military installations [24,49,50].

Race and/or ethnicity data were missing for approximately 5–7% of our cohort, and we were unable to precisely determine Filipino ancestry, which has been identified in previous reports as a risk factor, particularly for disseminated coccidioidomycosis [18]. Although missing race/ethnicity data are a limitation in our analysis, these data are generally more complete in the VHA compared with recent California and Arizona surveillance reports. These jurisdictions rely on laboratory-based reporting where race/ethnicity data are missing in one-third to one-half of reported cases [8,36,37]. These data are important since race and ethnicity are linked to self-rated health and comorbidities, healthcare utilization, and health disparities in both the management of chronic conditions and mortality among Veterans [51,52]. We used country of birth, where available, as a surrogate for Filipino ancestry, which is likely an underestimate since many Filipino Veterans are US-born. We did identify both Asian and Native Hawaiian/Pacific Islander race as being associated with coccidioidomycosis positivity; however, without additional chart review, we were unable to determine Filipino ancestry more precisely. Because country of birth was not available for all individuals, some Veterans born in the Philippines and Mexico were likely not captured. When reviewing hospitalizations within the first 45 days of testing, we did not review records to determine if patients were hospitalized with a respiratory infection or other complaints suggestive of coccidioidomycosis. For those that died within 30 days, we did not determine whether coccidioidomycosis was a principal or contributory cause of death. For future studies, we intend to further evaluate markers of disease severity and outcomes, including intensive care hospitalizations, rehabilitation stays, treatment, and causes of death.

## 5. Conclusions

This report highlights the epidemiology of coccidioidomycosis among VHA enrollees. During 2013–2022, testing and identification of US Veterans with coccidioidomycosis increased. Demographic features of patients testing positive for coccidiomycosis were consistent with previous reports, particularly among certain racial and ethnic groups. Rates of coccidioidomycosis for counties in Arizona and California followed similar trends compared with reported and confirmed cases over the period analyzed. As a national healthcare system report, this article provides a supplemental source of data on coccidioidomycosis in jurisdictions where coccidioidomycosis is not reportable and surveillance data are lacking or inconsistent. Providers should consider testing for coccidioidomycosis in patients with compatible histories who reside, work, or have travelled to areas where *Coccidioides* exists or may exist. Additional emphasis on targeted public health prevention strategies and education are needed to ensure at-risk patients are receiving timely testing, appropriate treatment, and follow-up.

## Figures and Tables

**Table 1:** Coccidioidomycosis-positive patients and rate per 100,000 Veterans in care, by year, Veterans Health Administration, 2014–2022.

Jurisdiction: VHA Count, Rate; [State/Co. Rate] *	2014	2015	2016	2017	2018	2019	2020	2021	2022	Av. Rate 2014–2022
**ARIZONA**	114, 79.6; [84.4]	204, 138.8; [112.8]	142, 94.6; [89.3]	166, 108.5; [98.8]	157, 99.9; [105.7]	172, 104.2; [144.1]	210, 124.4; [160.6]	206, 115.2; [157.7]	157, 87.6; [n/a]	105.9
Cochise	1, 14.4; [48.6]	3, 42.8; [38.0]	1, 14.1; [24.2]	2, 28.1; [24.1]	0, 0; [34.5]	1, 13.1; [61.2]	2, 26.2; [44.5]	0, 0; [52.2]	4, 49.1; [n/a]	20.9
Maricopa	63, 99.2; [98.2]	123, 186.9; [131.0]	61, 89.6; [106.2]	90, 129.8; [116.8]	76, 105.5; [128.0]	95, 126.8; [166.9]	115, 149.7; [185.2]	121, 147.7; [188.5]	82, 100.4; [n/a]	126.2
Mohave	0, 0; [26.5]	2, 24.5; [29.2]	3, 35.9; [23.3]	3, 35.1; [31.9]	5, 56.6; [39.9]	3, 32.8; [65.0]	4, 42.2; [88.3]	8, 80.3; [70.7]	1, 9.8; [n/a]	35.2
Pima	27, 88.8; [88.4]	42, 136.7; [119.0]	53, 172.4; [88.3]	50, 161.0; [99.6]	50, 160.7; [94.6]	47, 141.3; [136.9]	55, 164.7; [144.7]	47, 131.7; [123.2]	51, 143.3; [n/a]	144.5
Pinal	14, 165.4; [166.3]	25, 280.5; [161.4]	15, 160.3; [120.0]	18, 186.1; [121.1]	12, 116.6; [129.1]	18, 155.1; [204.3]	25, 204.8; [258.3]	19, 145.7; [225.9]	13, 97.8; [n/a]	168.0
Yavapai	2, 16.1; [18.6]	5, 39.8; [28.0]	3, 23.8; [25.0]	1, 7.8; [26.6]	5, 38.7; [31.0]	3, 22.7; [52.5]	3, 22.2; [43.0]	2, 14.6; [52.7]	1, 7.4; [n/a]	21.5
**CALIFORNIA**	90, 19.3; [6.0]	143, 30.3; [8.2]	136, 29.2; [14.2]	178, 38.3; [19.5]	157, 33.7; [19.2]	163, 33.9; [22.9]	126, 26.3; [18.2]	139, 27.2; [20.1]	120, 24.2; [n/a]	29.1
Contra Costa	1, 7.9; [2.7]	0, 0; [4.5]	2, 16.3; [6.0]	3, 24.8; [7.8]	5, 42.5; [9.5]	1, 7.4; [11.7]	1, 7.6; [12.5]	1, 6.9; [11.6]	1, 7.0; [n/a]	13.4
Fresno	5, 35.9; [16.6]	11, 77.4; [28.6]	8, 56.1; [62.1]	19, 132.3; [83.4]	10, 69.1; [63.7]	17, 110.3; [61.4]	6, 38.8; [43.6]	8, 49.8; [39.8]	12, 75.5; [n/a]	71.7
Kern	3, 33.5; [106.4]	25, 276.0; [122.9]	26, 286.3; [255.3]	30, 323.6; [312.8]	34, 360.6; [327.2]	25, 257.0; [372.0]	27, 278.4; [287.6]	13, 132.8; [306.2]	10, 101.0; [n/a]	227.7
Kings	2, 92.9; [71.9]	2, 91.0; [69.6]	6, 255.0; [158.0]	5, 207.8; [181.6]	5, 203.8; [113.3]	5, 194.8; [143.3]	2, 80.1; [101.5]	7, 268.3; [108.3]	2, 74.5; [n/a]	163.1
Los Angeles	25, 32.6; [4.0]	38, 49.7; [5.5]	25, 33.5; [7.2]	22, 29.9; [9.1]	30, 40.8; [9.9]	39, 52.0; [11.3]	36, 47.7; [10.6]	32, 39.3; [14.2]	20, 25.8; [n/a]	39.0
Monterey	0, 0; [5.6]	1, 15.3, [8.7]	0, 0; [18.0]	4, 61.0; [43.3]	0, 0; [53.9]	4, 60.3; [41.9]	0, 0; [25.2]	2, 28.4; [27.0]	3, 44.9; [n/a]	23.3
Orange	3, 12.7; [2.5]	1, 4.2; [5.5]	2, 8.6; [3.4]	6, 26.3; [7.2]	0, 0; [6.1]	4, 17.2; [9.0]	4, 16.9; [7.5]	2, 7.8; [8.8]	2, 8.2; [n/a]	11.3
Riverside	3, 8.0; [1.7]	11, 29.2; [2.4]	9, 23.8; [2.7]	10, 26.1; [5.6]	7, 17.9; [5.9]	8, 19.9; [11.9]	9, 21.4; [12.7]	8, 18.4; [18.4]	7, 16.6; [n/a]	20.1
Sacramento	2, 8.4; [1.2]	4, 16.5; [1.5]	7, 29.4; [1.8]	3, 12.4; [2.6]	2, 8.1; [2.8]	2, 7.7; [6.7]	0, 0; [3.6]	1, 3.6; [5.2]	2, 7.5; [n/a]	10.4
San Bernardino	3, 10.6; [1.6]	5, 17.2; [1.4]	6, 20.7; [1.8]	9, 31.4; [4.1]	6, 20.7; [ 4.5]	5, 16.7; [10.5]	6, 19.2; [10.7]	2, 6.3; [11.4]	7, 22.7; [n/a]	18.4
San Diego	14, 21.2; [2.7]	18, 26.6; [3.5]	15, 22.5; [4.0]	21, 31.3; [8.3]	13, 18.9; [8.3]	13, 18.1; [12.5]	10, 14.1; [13.8]	21, 27.2; [13.5]	24, 30.6; [n/a]	23.4
San Joaquin	3, 32.8; [8.5]	2, 21.9; [13.3]	3, 33.1; [25.9]	4, 44.9; [27.3]	6, 67.6; [32.0]	7, 76.4; [36.6]	3, 32.4; [17.3]	3, 31.2; [15.1]	2, 21.5; [n/a]	40.2
San Luis Obispo	2, 42.4; [9.9]	1, 21.4; [23.5]	5, 112.3; [93.2]	4, 91.0; [157.7]	7, 154.9; [124.4]	4, 87.6; [96.7]	3, 65.4; [64.5]	3, 66.0; [61.0]	0, 0; [n/a]	71.2
Santa Barbara	1, 18.2; [3.4]	3, 54.5; [5.6]	1, 18.9; [13.9]	2, 37.9; [25.7]	5, 93.4; [23.8]	2, 36.5; [16.6]	1, 18.6; [13.7]	4, 73.0; [14.6]	1, 18.3; [n/a]	41.0
Santa Clara	5, 32.2; [0.6]	1, 6.6; [0.9]	0, 0; [2.0]	6, 40.8; [2.0]	2, 14.1; [3.9]	0, 0; [4.0]	2, 14.3; [2.2]	0, 0; [3.3]	4, 28.0; [n/a]	15.1
Tulare	7, 125.7; [23.7]	8, 143.9; [25.9]	9, 164.8; [53.3]	15, 280.2; [61.6]	6, 109.3; [60.6]	7, 124.4; [87.5]	4, 70.8; [64.3]	11, 190.0; [65.8]	5, 89.4; [n/a]	144.4
Ventura	4, 44.5; [5.0]	2, 21.6; [5.7]	2, 22.7; [7.7]	0, 0; [30.3]	7, 77.1; [27.2]	6, 67.0; [43.9]	6, 66.5; [31.6]	6, 62.6; [21.3]	8, 87.4; [n/a]	49.9
**NEVADA**	8, 11.6	5, 7.0	8, 11.0	6, 8.0	13, 17.0	14, 17.5	16, 20.0	22, 25.6	12, 13.9	14.6
Clark	7, 16.2	4, 8.8;	7, 15.0	4, 8.3	12, 24.0	13, 24.6	14, 26.4	17, 29.6	12, 20.7	19.3
**TEXAS**	34, 7.6	29, 6.2	18, 3.8	21, 4.3	24, 4.8	43, 8.1	30, 5.6	21, 3.7	25, 4.3	5.4
Bell	3, 13.1	1, 4.1	1, 3.9	4, 14.8	2, 7.1	3, 10.1	1, 3.3	0, 0	0, 0	6.3
Bexar	8, 16.3	9, 17.3	3, 5.7	2, 3.7	7, 12.4	7, 11.2	13, 20.3	8, 11.1	11, 15.3	12.6
Dallas	1, 3.3	5, 16.3	3, 9.7	4, 12.8	4, 12.7	3, 9.6	1, 3.2	1, 3.1	1, 3.1	8.2
El Paso	8, 36.5	1, 4.4	2, 8.3	2, 8.2	2, 8.0	3, 11.5	0, 0	0, 0	3, 10.9	9.8
**WASHINGTON**	3, 2.4	3, 2.4	4, 3.1	3, 2.3	5, 3.7	2, 1.4	5, 3.6	8, 5.4	6, 4.2	3.2
King	0, 0	1, 5.0	2, 10.2	1, 5.1	0, 0	1, 5.1	0, 0	4, 18.3	1, 5.1	5.4

* Crude rates were calculated per 100,000 Veterans in care by state and county (based on Core Facility Uniques in VSSC Unique Patients Cube) by estimated date of onset using the earliest positive coccidioidomycosis test date. This table includes all US counties where there was at least 1 VHA coccidioidomycosis-positive case in each calendar year, or 4 or more coccidioidomycosis-positive cases in a single calendar year. Veterans in care data are tabulated by VHA fiscal year (1 October to 30 September each year). The 2014–2021 state and county rates for Arizona and California were pulled from published reports (2022 data not yet available) for comparison [36,37]. State/county rates not available for Nevada, Texas, or Washington. n/a = data not available.

**Table 2:** Characteristics of 67,526 Veterans tested for coccidioidomycosis, stratified by coccidioidomycosis test result, Veterans Health Administration, 2013–2022.

Variable N (%) or Median (Interquartile Range)	Positive Coccidioidomycosis Test	Negative Coccidioidomycosis Test	*p*-Value
**Total**	4204 (6.2%)	63,322 (93.8%)	
**Age**	**64 (16)**	**66 (16)**	**<0.001**
**Sex** Male Female	**3935 (93.6%)** **269 (6.4%)**	**57,870 (91.4%)** **5452 (8.6%)**	**<0.001** **<0.001**
**Race** White Black Asian American Indian/Alaskan Native Mixed Race Native Hawaiian/Pacific Islander Unknown	**2760 (65.6%)** **772 (18.4%)** **115 (2.7%)** **63 (1.5%)** 58 (1.4%) **81 (1.9%)** **355 (8.4%)**	**47,448 (74.9%)** **8385 (13.2%)** **893 (1.4%)** **694 (1.1%)** 697 (1.1%) **595 (0.9%)** **4610 (7.3%)**	**<0.001** **<0.001** **<0.001** **0.01** 0.09 **<0.001** **0.005**
**Ethnicity** Hispanic or Latino Not Hispanic or Latino Unknown	**540 (12.9%)** **3442 (81.9%)** 222 (5.3%)	**6290 (9.9%)** **53,915 (85.1%)** 3117 (4.9%)	**<0.001** **<0.001** 0.29
**Birth Country** Philippines Mexico	**41 (0.9%)** 32 (0.8%)	**435 (0.6%)** 424 (0.6%)	**0.03** 0.48
**Comorbidities** ^†^ Myocardial Infarction Congestive Heart Failure Peripheral Vascular Disease Cerebrovascular Disease Mild Liver Disease Chronic Obstructive Pulmonary Disease Rheumatic Disease Dementia Peptic Ulcer Disease Metastatic Solid Tumor Any Malignancy Hemiplegia or Paraplegia Renal Disease Moderate or Severe Liver Disease HIV/AIDS Diabetes Transplant Immunity Disorders	**370 (8.8%)** **458 (10.9%)** **664 (15.8%)** **588 (13.9%)** 561 (13.3%) **1605 (38.2%)** **237 (5.6%)** **114 (2.7%)** 189 (4.5%) **147 (3.5%)** **854 (20.3%)** **54 (1.3%)** **777 (18.5%)** 120 (2.9%) 139 (3.3%) 1511 (35.9%) 116 (2.8%) 87 (2.1%)	**7140 (11.3%)** **8727 (13.8%)** **12,554 (19.8%)** **11,031 (17.4%)** 8040 (12.7%) **32,609 (51.5%)** **4346 (6.9%)** **2484 (3.9%)** 3163 (4.9%) **3022 (4.7%)** **14,588 (23.0%)** **1095 (1.7%)** **13,046 (20.6%)** 1510 (2.4%) 1910 (3.0%) 21,977 (34.7%) 1890 (2.9%) 1216 (1.9%)	**<0.001** **<0.001** **<0.001** **<0.001** 0.22 **<0.001** **<0.001** **<0.001** 0.15 **<0.001** **<0.001** **0.03** **<0.001** 0.06 0.28 0.10 0.40 0.49
**County Incidence Rate** * High Medium Low	**1676 (39.9%)** **1118 (26.6%)** **1394 (33.2%)**	**19,013 (30.0%)** **14,867 (23.5%)** **29,236 (46.2%)**	**<0.001** **<0.001** **<0.001**
**Charlson Comorbidity Index (CCI) Score** 0 1–2 3–4 ≥5	**2 (4.3)** **1016 (24.2%)** **1210 (28.8%)** 812 (19.3%) **1166 (27.7%)**	**3 (5)** **10,299 (16.3%)** **19,957 (31.5%)** 12,835 (20.3%) **20,231 (31.9%)**	**<0.001** **<0.001** **<0.001** 0.13 **<0.001**

**^†^** Immunity Disorders and Transplant categories were based on Healthcare Cost and Utilization Project (HCUP) clinical classification software. All other comorbidities were grouped into major categories based on the Charlson Comorbidity Index (CCI) classifications. * High incidence rate (≥100 per 10,000 population), medium incidence (6–99.9 per 100,000 population), low incidence (<6 per 100,000 population).

**Table 3:** Association of age, sex, race, ethnicity, and select comorbidities with coccidioidomycosis positivity, Veterans Health Administration 2013–2022.

Risk Factor	Adjusted Risk Ratio (aRR) and 95% Confidence Interval (CI) *	*p*-Value
**Age**	**1.0005 (1.0004–1.0007)**	**<0.001**
**Sex** (Reference = Male) Female	**0.978 (0.972–0.984)**	**<0.001**
**Race (Reference = White)** Black Asian American Indian/Alaskan Native Mixed Race Native Hawaiian/Pacific Islander Unknown	**1.029 (1.022–1.036)** **1.060 (1.037–1.083)** **1.026 (1.004–1.048)** 1.020 (0.999–1.041) **1.068 (1.039–1.098)** **1.012 (1.003–1.021)**	**<0.001** **<0.001** **0.02** 0.06 **<0.001** **0.008**
**Ethnicity (Reference = Not Hispanic or Latino)** Hispanic or Latino Unknown	**1.021 (1.013–1.028)** 1.0004 (0.990–1.011)	**<0.001** 0.93
**Comorbidities (Reference = No Comorbidity)** HIV/AIDS Diabetes Transplant Immunity Disorders	0.997 (0.985–1.009) 1.004 (0.999–1.008) 0.993 (0.982–1.005) 1.009 (0.994–1.024)	0.68 0.051 0.26 0.23

* Log-binomial regression model evaluating relative risk of various risk factors with binary coccidioidomycosis test positivity compared to coccidioidomycosis test negativity. Model adjusted for age, sex, race, ethnicity, HIV/AIDS, diabetes, transplant, and immunity disorders.

**Table 4:** Association of sex, race, and ethnicity with disseminated coccidioidomycosis, Veterans Health Administration 2013–2022.

Risk Factor	Adjusted Risk Ratio (aRR) and 95% Confidence Interval (CI) *	*p*-Value
**Sex (Reference = Male)** Female	0.994 (0.977–1.012)	0.58
Race (Reference = White) Black Asian American Indian/Alaskan Native Mixed Race Native Hawaiian/Pacific Islander Unknown	**1.058 (1.037–1.081)** 1.015 (0.974–1.059) 0.995 (0.962–1.029) 1.033 (0.971–1.098) 1.032 (0.979–1.100) 0.995 (0.976–1.088)	**<0.001** 0.46 0.78 0.29 0.23 0.55
**Birth Country (Reference = Not Philippines)** Philippines	1.095 (0.973–1.231)	0.13
**Ethnicity (Reference = Not Hispanic or Latino)** Hispanic or Latino Unknown	**1.018 (1.0003–1.036)** 1.004 (0.979–1.029)	**0.04** 0.73

* Log-binomial regression model evaluating relative risk of various risk factors with binary disseminated coccidioidomycosis (including meningitis) compared to non-disseminated coccidioidomycosis. Model adjusted for sex, race, birth country, and ethnicity.

**Table 5:** Association of coccidioidomycosis-positive with all-cause mortality and hospitalization, Veterans Health Administration 2013–2022.

Outcomes	Coccidioidomycosis-Positive (N = 4204)	Coccidioidomycosis-Negative (N = 63,322)	Adjusted Risk Ratio (aRR) and 95% Confidence Interval (CI)	*p*-Value
Thirty-Day All-Cause Mortality *	109 (2.5%)	2428 (3.8%)	0.998 (0.996–1.001)	0.16
Forty-Five-Day Acute Care Hospitalization **	1499 (35.6%)	24,272 (38.3%)	0.999 (0.978–1.019)	0.92

* Log-binomial regression model evaluating relative risk of thirty-day all-cause mortality for binary coccidioidomycosis-positive patients compared to coccidioidomycosis-negative patients. Model adjusted for coccidioidomycosis positivity, CCI score, Black race. ** Log-binomial regression model evaluating relative risk of acute care hospitalization for binary coccidioidomycosis-positive patients compared to coccidioidomycosis-negative patients. Model adjusted for coccidioidomycosis positivity, CCI score, age (squared), sex, race, and ethnicity.

## Data Availability

The data that support the findings of this study are available from the corresponding author upon reasonable request.

## Supplementary Materials

The following supporting information can be downloaded at: https://www.mdpi.com/article/10.3390/jof9070731/s1. Figure S1: Demographic comparisons for coccidioidomycosis-positive (n = 4204) and coccidioidomycosis-negative (n = 63,322) patients, Veterans Health Administration, 2013–2022.
