# Supplementary material for: Epidemiology of Coccidioidomycosis in the Veterans Health Administration, 2013–2022
Source: J Fungi (Basel). 2023 Jul 6;9(7):731. doi: 10.3390/jof9070731 (PMC10381299; doi:10.3390/jof9070731)
Supplement: Supplementary file 1 [file jof-09-00731-s001.zip › jof-2455072-supplementary.pdf]

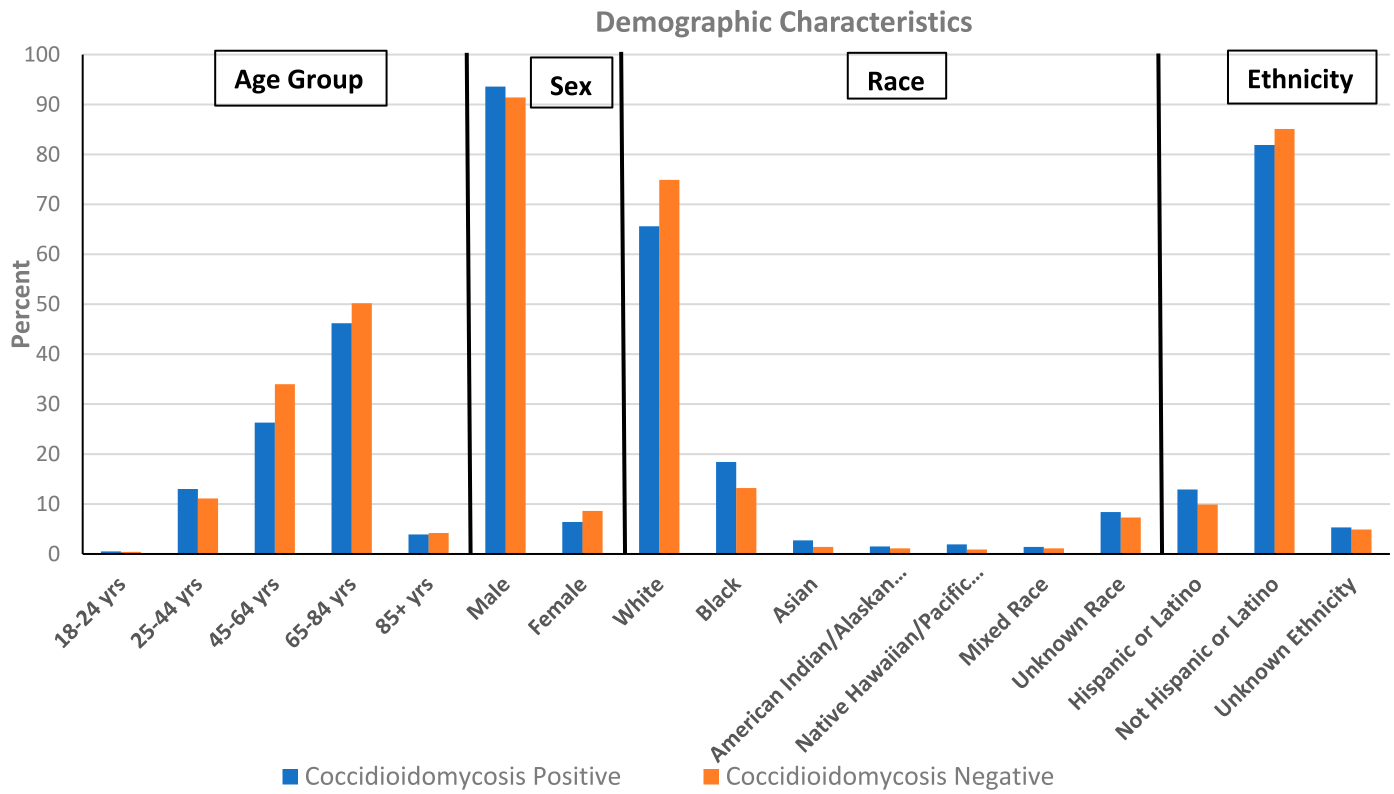

Figure S1. Demographic comparisons for coccidioidomycosis-positive (n = 4204) and coccidioidomycosis-negative (n = 63,322) patients, Veterans Health Administration, 2013–2022.
